# Infectious and obstetric determinants of anemia among pregnant women in Southwest Ethiopia

**DOI:** 10.3389/fgwh.2024.1421884

**Published:** 2024-09-19

**Authors:** Tewodros Yosef, Asaye Gizachew, Gossa Fetene, Desalegn Girma, Melsew Setegn, Aragaw Tesfaw, Binyam Girma Sisay, Nigusie Shifera

**Affiliations:** ^1^ School of Public Health, College of Medicine and Health Sciences, Mizan-Tepi University, Mizan Teferi, Ethiopia; ^2^ School of Medicine, Faculty of Health, Deakin University, Waurn Ponds, VIC, Australia; ^3^ Department of Midwifery, College of Medicine and Health Sciences, Mizan-Tepi University, Mizan-Teferi, Ethiopia; ^4^ School of Public Health, College of Medicine and Health Sciences, Debre Tabor University, Debre Tabor, Ethiopia; ^5^ Institute for Physical Activity and Nutrition, School of Exercise and Nutrition Sciences, Deakin University, Melbourne, VIC, Australia

**Keywords:** anaemia, malaria, birth interval, unsafe abortion, hookworm infection

## Abstract

**Background:**

Anaemia, characterized by low red blood cell or haemoglobin levels, impairs oxygen transport in the body and poses a major global public health issue, particularly affecting pregnant women and children. This study focuses on identifying the factors contributing to anaemia among pregnant women receiving antenatal care (ANC) at Mizan-Tepi University Teaching Hospital (MTUTH) in southwest Ethiopia.

**Methods:**

A hospital-based unmatched case-control study was conducted from July 1 to August 30, 2022, involving 370 pregnant women (90 with anaemia and 280 without). Data collection included questionnaires, laboratory tests (Hgb and stool examination), and anthropometric measurements. SPSS version 21 was used for data analysis, with binary logistic regression identifying factors associated with anaemia. The significance level was set at a *p-value <0.05*.

**Results:**

The study achieved a 100% response rate for both cases and controls. Factors identified as determinants of anaemia among pregnant women included malaria infection (AOR = 7.83, 95% CI: 3.89–15.8), hookworm infection (AOR = 2.73, 95% CI: 1.39–5.34), short birth interval (AOR = 7.11, 95% CI: 3.59–14.2), and history of unsafe abortion (AOR = 5.40, 95% CI: 2.46–11.8).

**Conclusion:**

This study found that malaria infection, hookworm infection, birth interval <33 months, and a history of unsafe abortion are factors contributing to anaemia in pregnant women. Strategies such as distributing insecticide-treated bed nets (ITNs) to combat malaria, improving sanitation, anthelmintic drugs, promoting family planning to prevent unwanted pregnancies and unsafe abortions, and providing preconception care can help reduce the incidence of anaemia.

## Introduction

Anemia, as defined by the World Health Organization (WHO), involves a decrease in red blood cell concentration or hemoglobin concentration, leading to reduced oxygen transport capacity (1). The definition of anemia in pregnant women is characterized by hemoglobin values less than 11.0 g/dl during the 1st and 3rd trimesters, and less than 10.5 g/dl in the 2nd trimester (2). Additionally, anemia can also be classified based on hematological indices such as mean corpuscular volume (MCV) less than 80fl, mean corpuscular hemoglobin concentration (MCHC) less than 30%, and mean corpuscular hemoglobin (MCH) less than 30 μg, indicating iron deficiency anemia (IDA) (3).

Globally, anemia affects 32.4 million (38.2%) pregnant women, with a prevalence of 14% in developed nations and 51% in developing ones (1). It's the most common pregnancy complication in developing countries (4, 5), particularly in South East Asia (48.7%) and Africa (46.3%) (1, 6). Anemia contributes to about 20% of maternal deaths, with around 510,000 annual deaths related to childbirth or early postpartum (7). In Sub-Saharan Africa, anemia prevalence is 35.6% among pregnant women (6), with Ethiopia at 32%, mainly due to poor nutrition, infections, menstrual blood loss, and frequent pregnancies (8).

Anemia is a major public health and nutritional issue, impacting health, social well-being, and economic development (9). It signals poor nutrition and health, leading to adverse pregnancy outcomes, maternal and child morbidity, and mortality. Risks include miscarriage, stillbirth, prematurity, low birth weight, and impaired child development (10). About 50% of anemia cases are due to iron deficiency, particularly in women of reproductive age due to increased iron needs during pregnancy, lactation, and menstruation (1, 11). Malaria infection is another significant cause of anemia (12). Malaria-induced anemia is multifactorial, caused by the destruction of infected RBCs, immune clearance of uninfected RBCs, splenic sequestration, reduced bone marrow RBC production, and oxidative damage from hemozoin (13, 14).

Anemia during pregnancy has multi-factorial causes, including modifiable and non-modifiable causes. The available studies showed that several factors that contribute to anemia during pregnancy such as socio-demographic & economic factors (age, religion, educational status, ethnicity, marital status, occupational status, and monthly family income), reproductive history (parity, gravidity, gestational age, family size, history of heavy menstrual cycle, birth interval, and history of contraceptive use), nutritional factors (food diversity, feeding habits, extra meals, and middle upper arm circumference), disease conditions (acute and chronic infections, parasitic infections, malaria and inherited or acquired disorders) and hygiene and sanitation factors (sources of water, using toilet, and hand-washing practice) are possible determinants of anemia (2, 12, 15–18).

Anemia is largely preventable and treatable if detected early. Effective management includes addressing underlying causes, restoring normal hemoglobin levels, and preventing complications (7). Despite government and stakeholder efforts in antenatal care, iron-folic acid supplementation, an deworming, anemia during pregnancy remains a public health issue in Ethiopia (19). Anemia in pregnancy is a major public health issue in Southwest Ethiopia (20), with severe implications for maternal and fetal health. Gudeta et al. (20) assessed the prevalence and factors of anemia among pregnant women in the study area through a cross-sectional study but did not explore infectious and obstetric factors linked to anemia.

In the study area, endemic parasitic infections such as malaria (21) and hookworms (22) are prevalent, and there is also a higher rate of unsafe abortions (23). This study aims to understand the determinants of anemia in this population, providing insights into infectious and obstetric factors. The findings will help policymakers and healthcare providers implement effective, evidence-based interventions to improve maternal and child health outcomes in the region.

## Methods and materials

### Study design, setting, and period

A hospital-based unmatched case-control study was conducted among pregnant women attending ANC at MTUTH in southwest Ethiopia. MTUTH, affiliated with Mizan-Tepi University since 2016, serves a catchment area of over two million people, including Kaffa, Shaka, West Omo, and parts of the Gambella region, located 585 km southwest of Addis Ababa. The hospital offers various care units, an emergency ward, and wards for medical, pediatric, surgical, gynecology, and obstetrics, with two operation rooms. The study was conducted from July 1 to August 30, 2022.

### Populations

The source population comprised all pregnant women in southwest Ethiopia. The study population consisted of pregnant women attending antenatal care at MTUTH. Cases were defined as pregnant women with anemia (low Hgb levels), while controls were non-anemic pregnant women. The inclusion criteria were pregnant women attending antenatal care at Mizan-Tepi University Teaching Hospital (MTUTH) who were willing provide informed consent.

### Sample size determination and sampling technique

The sample size was calculated using Epi Info 7.2 with assumptions of a 17% prevalence of chronic illness among anemic pregnant women (18), a 95% confidence interval, 80% power, and a 1:3 case-control ratio. Initially calculated at 336, the total study participants adjusted for a 10% non-response rate, resulting in 370 participants, including 90 cases and 280 controls.

Cases were consecutively sampled until the required sample size was achieved. For each case, three controls were selected on the same day from the obstetrics and gynecology clinic, where they sought antenatal care.

### Data collection tools, procedures, and quality control

Data was collected using a questionnaire, laboratory tests (Hgb and stool examination), and anthropometric measurements. The questionnaire, initially developed in English, was translated into Amharic and back-translated to ensure consistency. Data was collected through face-to-face interviews using the Amharic version of the questionnaire. The questionnaire covered sociodemographic details (age, residence, marital status, education, occupation), hygiene, nutrition, and health factors (water sources, latrine availability, raw vegetable consumption, tea or coffee consumption post-meal, iron-folic acid supplementation, malaria history), and obstetrics and gynecology factors (gestational age, parity, history of unsafe abortion, number of children, birth interval). Midwives assessed nutritional status using mid-upper arm circumference (MUAC). Two skilled laboratory technicians used HemoCue to measure hemoglobin levels and conducted stool examinations to identify hookworm infection. Capillary blood samples were taken using sterile techniques, and hemoglobin levels were measured instantly with HemoCue photometers.

Stool samples were collected from each participant using a clean, wide-mouthed, leakproof stool cup. A stool wet mount was prepared with saline and/or iodine and examined microscopically within 30 min of collection to identify hookworm infection. A pretest with 5% of the sample was conducted at Chena hospital before data collection. Comprehensive training was also provided to data collectors and supervisors to ensure consistent and clear data collection. Two medical lab technicians and two midwives from the ANC clinic were trained and tasked with data collection.

### Study variables

The dependent variable was anemia. The independent variables were socio-demographic & economic (age, residence, education, marital status, and occupation), gynecology and obstetrics (gestational age, parity, gravidity, history of unsafe abortion, number of children, and birth interval), hygiene, diseases & nutrition-related factors [nutritional status, hookworm infection, malaria history, iron supplementation, hot drinks after meal (tea or coffee), eating raw vegetables].

### Operational definitions

Anemia during pregnancy is classified based on WHO guideline: Hgb levels ≤11 g/dl in the first and third trimesters, and <10.5 g/dl in the second trimester indicate anemia (1).

Gestational age is calculated from the last normal menstrual period (LNMP) and categorized into trimesters: first trimester (below 14 weeks GA), second trimester (14–27 weeks GA), and third trimester (above 28 weeks GA) (1).

Nutritional status is assessed using middle upper arm circumference (MUAC): <22 indicates undernutrition, while ≥23 indicates normal (24, 25).

Iron supplementation involves daily intake of ferrous sulfate, ferrous fumarate, or ferrous gluconate tablets at a minimum recommended dose for 90 days (8).

The WHO defines unsafe abortion as a pregnancy termination before 28 weeks of gestation or before the fetus is viable, carried out by unskilled individuals or in environments lacking minimal medical standards, or both (26).

A short birth interval is less than 33 months after the previous live birth, while an optimal birth interval is 33 months or more between consecutive births (27, 28).

### Data processing and analysis

Data were entered using Epi-data version 3.1 and exported to SPSS version 21 for analysis. Descriptive statistics were used to analyze participant characteristics and covariates associated with anemia. Continuous variables were assessed for normality to determine summary measure mean and standard deviation (SD) for normally distributed variables, and median with interquartile range for non-normally distributed ones. Categorical variables were summarized using frequencies and percentages. Binary logistic regression assessed the association between independent variables and anemia. Independent variables with *P < 0.25* in the bi-variable analysis were selected for multivariable logistic regression analysis. Multivariable logistic regression analysis was conducted to identify factors significantly associated with the dependent variable. Results were reported using adjusted odds ratio (AOR) with 95% confidence intervals. The Hosmer-Lemeshow goodness of fit test indicated the model was well-fitted (*P-value = 0.269*). No significant interactions were detected. A significance level was set at *P-value <0.05*.

## Results

### Socio-demographic characteristics

Three hundred seventy (370) pregnant women participated in the study, with 280 controls and 90 cases, resulting in a 100% response rate. Among them, 35.6% of cases and 43.2% of controls had no formal education. Additionally, 18.9% of cases and 26% of controls were government employees, while 13.3% of cases and 18.6% were farmers (Table 1).

**Table 1:** Socio-demographic characteristics of study participants at MTUTH in southwest Ethiopia. Data are presented as mean (± SD) and *n* (%).

Variables	Categories	Cases	Controls
Age [26.7 ± 5.8] years	15–19	13 (14.4)	24 (8.60)
	20–24	30 (33.3)	96 (34.2)
	25–29	23 (27.8)	99 (35.4)
	≥ 30	22 (24.4)	61 (21.8)
Residence	Rural	36 (40.0)	142 (50.7)
	Urban	54 (60.0)	138 (49.3)
Marital status	Currently unmarried	6 (6.70)	14 (5.0)
	Currently married	84 (93.3)	266 (95.0)
Education level	No formal education	32 (35.6)	121 (43.2)
	Formal education	58 (64.4)	159 (56.8)
Occupation	Housewife	19 (21.1)	71 (25.4)
	Merchant	27 (30.0)	66 (23.6)
	Government employee	17 (18.9)	73 (26.0)
	Farmer	12 (13.3)	52 (18.6)
	Others^a^	15 (16.7)	18 (6.40)

^a^ Others, daily laborers and students.

### Hygiene, nutritional and disease related characteristics

Forty-three (47.8%) of cases and 147 (52.5%) of controls used a public piped water source. Sixty-five (72.2%) of cases and 116 (41.4%) of controls had experienced malaria infection in the past 12 months. Furthermore, 38.9% of cases and 20.4% of controls had hookworm infection (Table 2).

**Table 2:** Hygiene, nutritional, and disease factors of study participants at MTUTH in southwest Ethiopia. Data are presented as *n* (%).

Variables	Categories	Cases	Controls
Public piped water	Yes	43 (47.8)	147 (52.5)
	No	47 (52.2)	133 (47.5)
Latrine availability	Yes	85 (94.4)	262 (93.6)
	No	5 (5.60)	18 (6.40)
Consuming raw vegetables	Yes	42 (46.7)	116 (41.4)
	No	48 (53.3)	164 (58.6)
Drinking tea or coffee right after a meal	Yes	50 (55.6)	114 (40.7)
	No	40 (44.4)	166 (59.3)
Iron supplementation	Yes	32 (35.6)	180 (64.3)
	No	58 (64.4)	100 (35.7)
Undernutrition (using MUAC)	Yes	55 (61.1)	196 (70.0)
	No	35 (38.9)	84 (30.0)
Malaria history	Yes	65 (72.2)	116 (41.4)
	No	25 (27.8)	164 (58.6)
Hookworm infection	Yes	35 (38.9)	57 (20.4)
	No	55 (61.1)	223 (79.6)

### Gynecology and obstetrics related characteristics

Sixty-three (70%) of cases and 185 (66.1%) of controls were multigravida. Moreover, 32.2% of cases and 8.6% of controls had a history of abortion, and 46.7% of cases and 8.9% of controls had a short birth interval (Table 3).

**Table 3:** Obstetric and gynecologic factors of study participants at MTUTH in southwest Ethiopia. Data are presented as median (IQR) and *n* (%).

Variables	Categories	Cases	Controls
Current gestational age	First trimester	35 (38.9)	85 (30.4)
	Second trimester	23 (25.6)	90 (32.1)
	Third trimester	32 (35.5)	105 (37.5)
Gravidity	Primigravida	27 (30.0)	95 (33.9)
	Multigravida	63 (70.0)	185 (66.1)
Parity	Nullipara	29 (32.2)	94 (33.6)
	Primipara	30 (33.3)	90 (32.1)
	Multipara	31 (34.4)	96 (34.3)
History of unsafe abortion	Yes	29 (32.2)	24 (8.60)
	No	61 (67.8)	256 (91.4)
Number of children [4 (1–5)]	<3	65 (72.2)	211 (75.4)
	≥3	25 (27.8)	69 (24.6)
Birth interval	Short	42 (46.7)	25 (8.90)
	Optimal	48 (53.3)	255 (91.1)

### Determinants of anemia

After controlling for confounding variables, factors such as a history of malaria infection (AOR = 7.83, 95% CI: 3.89–15.8), hookworm infection (AOR = 2.73, 95% CI: 1.39–5.34), short birth intervals (AOR = 7.11, 95% CI: 3.59–14.2), and a history of abortion (AOR = 5.40, 95% CI: 2.46–11.8) were identified as significant determinants of anemia (Table 4).

**Table 4:** Determinants of anemia among study participants at MTUTH in southwest Ethiopia.

Variables	Categories	Cases	Controls	COR, 95% CI	AOR, 95% CI
Residence	Rural	36	142	0.65 (0.41, 1.05)*	0.71 (0.39, 1.28)
	Urban	54	138	1	1
Drinking tea or coffee right after a meal	Yes	50	114	1.82 (1.13, 2.94)**	1.33 (0.73, 2.44)
	No	40	166	1	1
Undernutrition (using MUAC)	Yes	55	196	1.48 (0.78, 2.16)*	1.65 (0.87, 3.09)
	No	35	84	1	1
Malaria history	Yes	65	116	3.68 (2.19, 6.18)**	7.83 (3.89, 15.8)**
	No	25	164	1	1
Hookworm infection	Yes	35	57	2.49 (1.49, 4.16)**	2.73 (1.39, 5.34)*
	No	55	223	1	1
History of unsafe abortion	Yes	29	24	5.07 (2.71, 9.32)**	5.40 (2.46, 11.8)**
	No	61	256	1	1
Birth interval	Short	42	25	8.93 (4.98, 15.9)**	7.11 (3.59, 14.2)**
	Optimal	48	255	1	1

1 = reference value.

* *p-value* <0.25.

** *p-value* <0.05; COR, crude odds ratio; AOR, adjusted odds ratio; CI, confidence interval.

## Discussion

This study at Mizan-Tepi University Teaching Hospital in southwest Ethiopia aimed to uncover factors influencing anemia among pregnant women. It identified malaria and hookworm infections, short pregnancy intervals, and previous unsafe abortion as significant contributors to anemia. These findings emphasize the need for targeted interventions addressing infectious diseases and reproductive health to reduce anemia among pregnant women in the region.

Malaria infection was significantly associated with anemia in pregnant women. The odds of anemia among pregnant women with malaria were 7.8 times higher than among those without malaria infection. This finding aligns with studies conducted in Ethiopia and Sudan (3, 29, 30). The association may be attributed to malaria's impact on red blood cells (RBCs), as the infection accelerates RBC destruction faster than the body can replace them. Malaria causes increased destruction and removal of both infected and uninfected erythrocytes, decreases erythrocyte production, and suppresses the erythropoietin response, all contributing to malarial anemia (14, 31).

Hookworm infection was significantly associated with anemia in pregnant women. Pregnant women with a hookworm infection were 2.7 times more likely to develop anemia compared to those without such an infection. This finding is consistent with studies conducted in Ethiopia (3, 17, 29, 32). Hookworms attach to the upper intestinal mucosa and ingest blood, causing gastrointestinal blood loss and depletion of iron, folic acid, and vitamin B12, which leads to anemia. Additionally, hookworm infection not only causes blood loss and competition for nutrients but also results in loss of appetite, decreased intestinal motility, and damage to the digestive tract, leading to malabsorption of nutrients (33).

Pregnant women with a history of unsafe abortion had 5.4 times higher odds of developing anemia compared to those without such a history. This finding aligns with studies conducted at public hospitals in Ethiopia (17, 29). The risk of anemia following an unsafe abortion is heightened due to significant blood loss and inadequate time for the body to restore iron levels (34). The combined effects of blood loss, iron deficiency, and the body's compromised state post-abortion highlight the critical need for comprehensive medical and nutritional support for women with a history of unsafe abortion to prevent anemia and promote overall health.

Pregnant women with a short birth interval face seven times higher odds of developing anemia compared to those with an optimal birth interval. This finding is consistent with a study conducted in Harar, Ethiopia (35). A short birth interval increases the risk of adverse maternal outcomes, such as pregnancy-related anemia. This heightened risk stems from inadequate time for the mother to recover from the nutritional demands and physical stress of the previous pregnancy. Insufficient intervals do not allow adequate replenishment of essential nutrients like iron, which are depleted during pregnancy and breastfeeding. These deficiencies can accumulate and lead to anemia (36). Therefore, ensuring adequate spacing between pregnancies is crucial for maternal health, allowing time for recovery and reducing the risk of anemia and other related complications.

### Strengths and limitations of the study

The study demonstrated a notable strength with a 100% response rate. Other strengths included a robust study design, the use of multiple methods (questionnaires, lab tests, anthropometric measurements) for data collection, and high data quality maintained through pretesting, comprehensive training, and standardized techniques. The study's limitations include its hospital-based setting, which may not fully represent the broader community and thus limits generalizability. The small sample size further restricts the applicability of the findings to a larger population due to reduced statistical power and reliability. Additionally, self-reported data could be subject to recall bias, and the study did not account for other potential factors associated with anemia, such as genetic disorders.

## Conclusion

This study identified malaria infection, hookworm infection, birth interval <33 months, and a history of unsafe abortion as key factors contributing to anemia among pregnant women. To prevent anemia, collaboration among healthcare providers, government health institutions, and stakeholders is essential. Strategies include promoting insecticide-treated bed nets to combat malaria, cleaning swampy areas to reduce mosquito breeding grounds, provision of anthelmintic drugs to prevent hookworm infection, and providing comprehensive family planning services to prevent unplanned pregnancies. Additionally, offering preconception care and expanding access to modern family planning methods can help reduce short birth intervals, thereby improving maternal health outcomes and effectively addressing the burden of anemia in pregnant women.

## Data Availability

The raw data supporting the conclusions of this article will be made available by the authors, without undue reservation.
